# 

**Published:** 1908-07-25

**Authors:** 


					,.;? 41>t;:.k L ? uf' ''THE MODERN
Y?lsSr*?iile Address I , I 2734 Gerrard.
??Dejiale, LqlWdda. 3~ j"1'MEDICAL JOURNAL. || .?
NeW SERIES. No. 72, Vol. III. CUrrrriiriAv
ia^J?o.lR4?-.M-XUV.  - - -SATURDAY,
July 25th, iy08.
PRICE THREEPENCE

				

